# Exclusion of the endoplasmic reticulum from the *C. elegans* meiotic spindle controls spindle size

**DOI:** 10.17912/micropub.biology.001812

**Published:** 2025-09-19

**Authors:** Alma Aquino, Francis McNally

**Affiliations:** 1 Molecular and Cellular Biology, University of California, Davis, Davis, California, United States

## Abstract

The nuclear envelope is composed of sheet-like ER that separates chromatin from tubulin during interphase. During oocyte maturation in *C. elegans* , a fenestrated envelope of sheet-like ER continues to envelope the meiotic spindle through metaphase I. ER is thus excluded from the nuclear/spindle volume during spindle assembly. To test the importance of this exclusion, we forced ER into the meiotic spindle by coupling kinesin motor domains to the ER. Forcing ER into the spindle interior caused a statistically significant increase in metaphase spindle width. Exclusion of ER from the spindle thus affects spindle geometry.

**Figure 1. Optogenetically attaching kinesin motor heads to the ER leads to the ER invading the meiotic spindle space, increasing spindle width.**
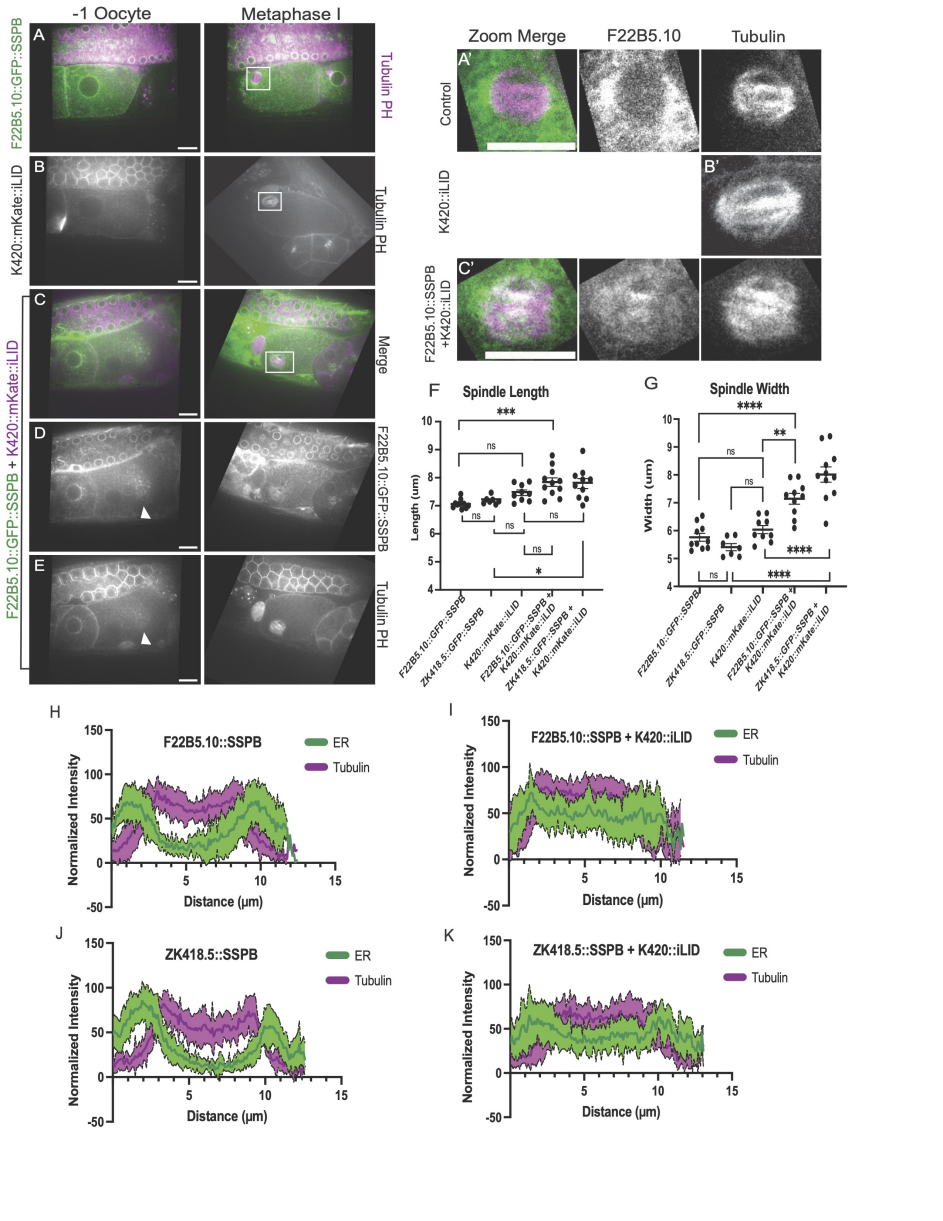
A-E. Representative images from time-lapse sequence of F22B5.10 (ER)::GFP::SSPB (green) and mKate tubulin (red) in -1 oocytes and metaphase I meiotic embryos. Magnified images of the spindle region are shown in A'-C'. F. Metaphase I spindle length measurements. G. Metaphase I spindle width measurements. H-K. Normalized fluorescence intensity along a 5 pixel wide line drawn down the length of the metaphase I spindle. Red = mKate::tubulin. Green = GFP::ER. Solid line is the mean of 10 spindles. Shaded region is the standard error of the mean of 10 spindles. Bar: 10um. ns not significant, * p<0.05, ** p<0.01, *** p< 0.001, **** p<0.0001 One-way ANOVA with Tukey's post hoc multiple comparisons test conducted with Graphpad Prism.

## Description

During interphase, the nuclear envelope separates tubulin from chromatin thus providing one universal mechanism preventing spindle assembly before DNA replication. Upon entry into M-phase in many cell types, a remnant of the nuclear envelope consisting of fenestrated ER sheets continues to envelope the mitotic spindle as it assembles, allowing entry of tubulin dimers but excluding ER and other membranes from the spindle (Lu et al., 2009; Smyth et al., 2012; Schweizer et al., 2015). ER sheets also envelope the *C. elegans* oocyte meiotic spindle as it assembles and ER, mitochondria, and yolk granules are all excluded from the nuclear/spindle volume through metaphase I (Gong et al. 2024). The significance of excluding ER and other organelles from the spindle interior has been difficult to test. In Hela cells, expression of a non-phosphorylatable STIM1 caused penetration of ER into the mitotic spindle but no effect on spindle structure or function was demonstrated (Smyth et al., 2012).

Here we caused penetration of the ER into the *C. elegans* metaphase I female meiotic spindle by optogenetically coupling kinesin motor domains to either of two transmembrane proteins of the ER, ZK418.5 (homologous to human TMEM147) or F22B5.10 (homologous to human TMCO1). iLID and SSPB are proteins engineered to bind with higher affinity when exposed to blue light (Guntas et al., 2015). Co-expression of K420::mKate::iLID (kinesin motor domains with no cargo-binding tail) with ZK418.5 ::GFP::SSPB was previously shown to transiently pack ER into the center of the most mature prophase-arrested oocytes adjacent to the spermatheca (-1 oocytes) (Aquino et al., 2025) because microtubule plus ends are thought to extend inward from the plasma membrane. Co-expression of K420::mKate::iLID with F22B5.10 ::GFP::SSPB also caused inward packing of ER in -1 oocytes ( Fig. 1D arrowhead). Co-expression of either of these transgene pairs also caused the ER to invade the metaphase I spindle volume ( Fig. 1A '-C', Fig. 1H- 1K). Neither transgene pair caused a significant reduction in hatch rates (Aquino et al., 2025) indicating that meiosis proceeded somewhat normally. Expression of each transgene pair caused a significant increase in spindle width ( Fig. 1G ) but not length ( Fig. 1F ) relative to each transgene alone. This result indicates that failure to exclude ER from the spindle volume causes a change in spindle geometry that is not essential for meiotic progression. Because we did not monitor chromosomes and because trisomies are viable in *C. elegans* (Gong and McNally, 2023), we cannot conclude whether meiotic chromosome segregation was completely normal.

## Methods

Optogenetic chimaeras were assembled with germline-optimized (Fielmich et al., 2018), versions of GFP, mKate, SSPB and iLID, and PCR amplified genomic regions of UNC-116 , F22B5.10 and *mex-5* promoter and *tbb-2* 3'UTR in the RMCE vector pLF3FShC (Nonet, 2020) as described previously (Aquino et al., 2025). K420::mKate::iLID was inserted at the jsTi1490 chromosome IV landing site in NM5176 . All SSPB transgenes were inserted at the jsSi1579 chromosome II landing site in NM5402 and FLP recombinase was removed by outcrossing.

For time-lapse imaging, anesthetized worms were mounted between an agarose pad and coverslip as described in (Danlasky et al., 2020) and subjected to single plane time-lapse imaging on a Yokogawa CSU10 spinning disk confocal microscope equipped with an Olympus 100X 1.3 PlanApo objective and a Hammamatsu Orca Quest qCMOS detector. Exposures were captured every 5 seconds. Metaphase spindle length and width measurements were collected on spindles before the initiation of shortening.

ER enrichment at the poles and exclusion from the meiotic spindle in Figs. 1H-1K was documented by displaying GFP fluourescence intensity along a 5 pixel wide line drawn down the length of the spindle with the line tool on Image J (FIJI). The data was then normalized to have the brightest pixel equal 100 while the lowest pixel would be 0. The fluorescence intensity of the meiotic spindle was measured in a similar fashion but displaying the normalized fluorescence intensity of mKate::tubulin. The solid lines in Fig. 1H- 1K represent the mean intensity from 10 spindles and the colored shaded regions indicate the SEM from 10 spindles.

## Reagents

*C. elegans Strains*

**Table:** 

Strain #	Genotype	Figure
FM1010 ( F22B5.10 )	* duSi29[pFM1994 ( F22B5.10 ::GFP::SSPB(nanoGLO))] II; * * duSi14[pFM1953(K420::mKateGLO::iLID)]IV; ItIs44pAA173 [pie-1p-mCh::PH(PLC1delta1) + unc-119 (+)]V; wjIs76 [Cn_ unc-119 (+) pie-1p::mKate2:: tba-2 ] *	Figure 1C- 1E and 1C'
FM1043	*duSi14[pFM1953 (K420::mKate-GLO::iLID)IV;* * ItIs44pAA173 [pie-1p-mCh::PH(PLC1delta1) + unc-119 (+)]V; wjIs76 [Cn_ unc-119 (+) pie-1p::mKate2:: tba-2 ] *	Figure 1B and 1B'
FM1135 ( ZK418.5 )	* duSi31[pFM1968 ( ZK418.5 ::GFP::SSPB(nanoGLO)]II; duSi14[pFM1953(K420::mKateGLO::iLID)]IV; ItIs44pAA173 [pie-1p-mCh::PH(PLC1delta1) + unc-119 (+)]V; wjIs76 [Cn_ unc-119 (+) pie-1p::mKate2:: tba-2 ] *	Figure 1K
FM1221 ( F22B5.10 )	* duSi29[pFM1994; F22B5.10 ::GFP(GLO)::SSPB(nanoGLO)]II; ItIs44pAA173 [pie-1p-mCh::PH(PLC1delta1) + unc-119 (+)]V; wjIs76 [Cn_ unc-119 (+) pie-1p::mKate2:: tba-2 ] *	Figure 1A and 1A'
FM1267 ( ZK418.5 )	* duSi31[pFM1968 ( ZK418.5 ::GFP::SSPB(nanoGLO)]II; ItIs44pAA173; [pie-1p-mCh::PH(PLC1delta1) + unc-119 (+)]V; wjIs76 [Cn_ unc-119 (+); pie-1p::mKate2:: tba-2 ] *	Figure 1J
